# Unintended Medial Cord Block Following Pectoral Nerve (PECS) I and II Blocks: A Case Report Highlighting Anatomical Considerations and Technical Optimization

**DOI:** 10.7759/cureus.105793

**Published:** 2026-03-24

**Authors:** Shreya Desai, Alejandro Hallo-Carrasco, Adarsh Menon, George Holan, Alexander Pekurovsky

**Affiliations:** 1 Anesthesiology, New Jersey Medical School, Rutgers University, Newark, USA; 2 Office of Education, New Jersey Medical School, Rutgers University, Newark, USA; 3 Anesthesiology, Cooperman Barnabas Medical Center, Livingston, USA

**Keywords:** brachial plexus, clavipectoral fascia, medial cord, pecs block, regional anesthesia

## Abstract

The pectoral nerve (PECS) block is a relatively newer regional anesthetic technique that provides analgesia to the upper anterior chest wall. It is commonly used for breast surgery and has also been applied to a range of other procedures, from pacemaker insertion to anterior shoulder surgeries. PECS blocks provide effective pain relief while avoiding more invasive techniques such as paravertebral blockade, making them an appealing option for perioperative analgesia. Although generally considered safe, rare complications may occur if the local anesthetic spreads beyond its intended fascial planes, potentially affecting the brachial plexus.

We report the case of a 35-year-old woman who underwent bilateral augmentation mammoplasty under general anesthesia, with ultrasound-guided PECS I and II blocks administered postoperatively at the conclusion of the procedure (15 mL per plane of a mixture of liposomal and standard bupivacaine). Approximately one hour after surgery, she developed right upper extremity paresthesia, motor weakness, and sensory deficits consistent with medial cord (C8-T1) involvement. She was managed conservatively with protective measures and counseling, with complete resolution of symptoms by postoperative day 6. This case highlights the possibility that surgical disruption of pectoral fascial planes, together with injectate volume, may permit unintended proximal spread of local anesthetic and underscores the importance of meticulous anatomical technique.

## Introduction

The pectoral nerve (PECS) I and II nerve blocks have gained widespread adoption in diverse surgical procedures due to their technical simplicity, ultrasound-guided precision, and favorable safety profile. These interfascial plane blocks differ in depth and targets: the PECS I block is performed between the pectoralis major and minor muscles and primarily targets the PECS, whereas the PECS II block is performed in a deeper plane between the pectoralis minor and serratus anterior muscles, extending coverage to the lateral branches of the thoracic intercostal nerves (T2-T6) and the lateral thoracic wall [1].

Although these blocks are considered relatively superficial and low risk, complications may occur when local anesthetic spreads beyond the intended fascial planes [2]. Neurologic complications are rare, and brachial plexus involvement has been described only in isolated case reports, highlighting the uncommon nature of this presentation. One such clinically significant complication is the possible involvement of the medial cord of the brachial plexus, which originates from the anterior division of the lower trunk (C8-T1). This may result in weakness of the intrinsic hand muscles, impaired grip strength, and sensory deficits in the ulnar distribution, potentially impacting hand function and patient quality of life.

In this report, we describe a rare case of neurologic findings consistent with medial cord involvement following combined PECS I and II blocks. By analyzing our case alongside the few similar reports available in the literature, we aim to highlight a potentially underrecognized mechanism of complication and propose a hypothesis regarding the anatomical pathways that may facilitate anesthetic spread to the brachial plexus. This discussion underscores the critical role of detailed anatomical knowledge and technical precision in preventing such complications.

This manuscript was prepared in accordance with the Case Report (CARE) guidelines, which are freely available and do not require licensing [3]. The CARE checklist has been included as supplementary material, and an additional reference to the Enhancing the Quality and Transparency of Health Research Network (EQUATOR) has been provided to facilitate access [4]. This manuscript adheres to the relevant EQUATOR reporting standards. In accordance with institutional policy, only non-identifiable patient information was included, and written informed consent for publication was obtained from the patient.

## Case presentation

An otherwise healthy 35-year-old woman presented for bilateral augmentation mammoplasty under general anesthesia with endotracheal intubation. Her past medical and surgical histories were unremarkable. The surgical procedure, performed using a subpectoral (submuscular) approach, was uneventful, and the patient was transferred to the post-anesthesia care unit (PACU) in stable condition.

**Figure 1:**
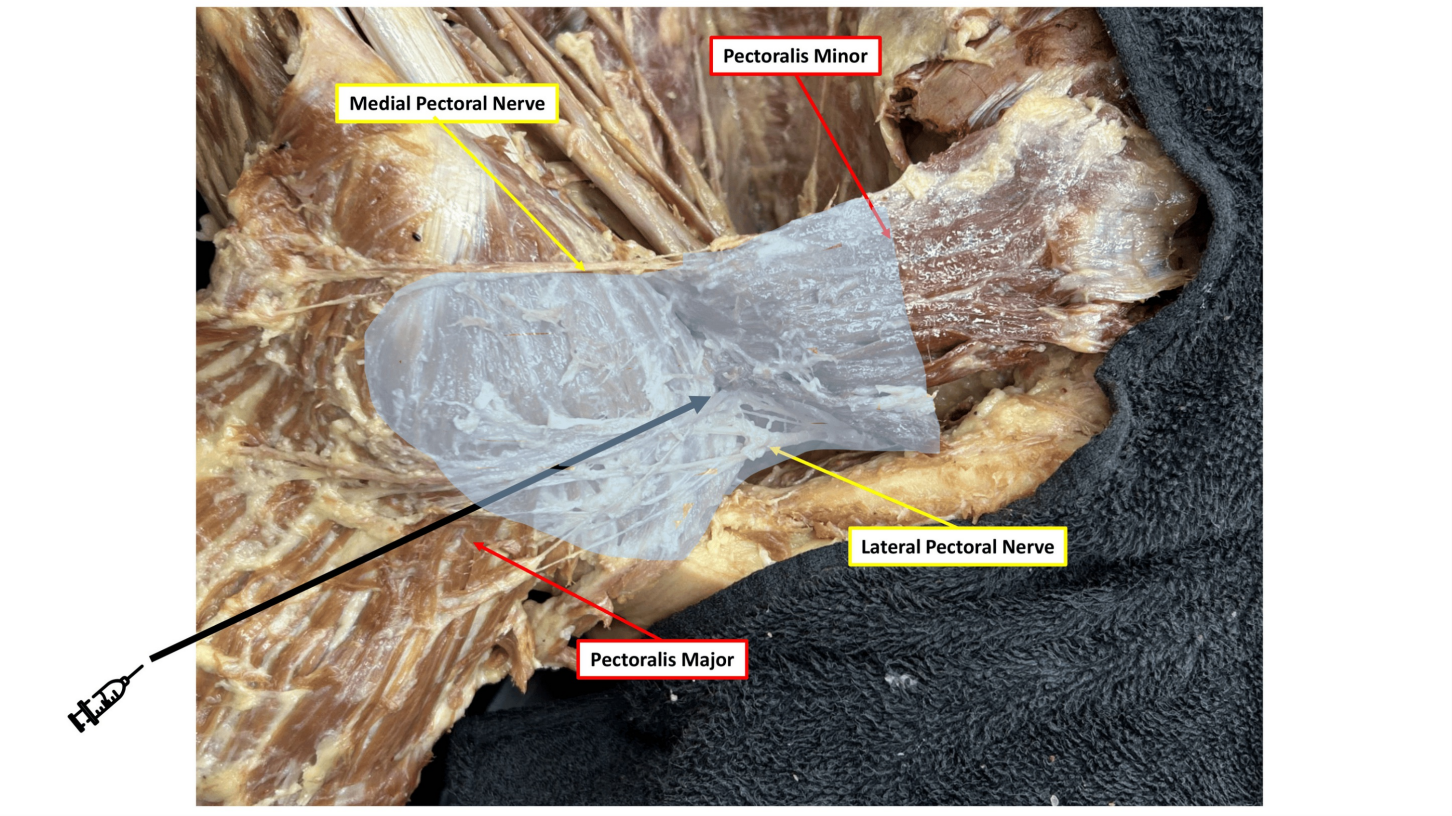
Cadaveric visualization of the PECS I plane between the pectoralis major and minor Cadaveric dissection demonstrating the interpectoral (PECS I) fascial plane located between the pectoralis major and pectoralis minor muscles. The lateral and medial PECS are shown traversing this compartment. The shaded region illustrates the target injectable space for the PECS I block, corresponding to the site where the local anesthetic was deposited in the clinical case. Images were obtained from an adult human cadaver donated to the Rutgers New Jersey Medical School Anatomical Donation Program and used in accordance with institutional educational and ethical guidelines. PECS: pectoral nerve Image Credit: Authors, using Microsoft PowerPoint (Microsoft Corporation, Redmond, Washington, USA)

For multimodal analgesia, ultrasound-guided bilateral PECS I and II blocks were performed postoperatively (Figure 1). The PECS II injection was performed first, followed by PECS I after withdrawal of the needle to a more superficial plane. This was in addition to intravenous acetaminophen, nonsteroidal anti-inflammatory drugs (NSAIDs), and opioids as needed for breakthrough pain. The patient tolerated the procedure well without immediate complications.

A solution was prepared by mixing 20 mL of liposomal bupivacaine (Exparel™, 1.3%), 20 mL of 0.25% bupivacaine, and 20 mL of normal saline to achieve the target concentration. Fifteen mL of the prepared solution was injected between the pectoralis major and minor muscles for the PECS I block. The PECS II block involved administration of an additional 15 mL of the same solution between the pectoralis minor and serratus anterior muscles. The patient reported effective analgesia in the appropriate dermatomes.

Approximately one hour after block administration, the patient reported new-onset paresthesia in the right upper extremity. Physical examination revealed motor weakness predominantly affecting the intrinsic hand muscles, including finger abduction/adduction and grip strength, as well as reduced thumb opposition. Wrist flexion was mildly decreased, while wrist extension, elbow flexion, and elbow extension were preserved. Sensory deficits were noted in the ulnar distribution, involving the fifth digit, the medial half of the fourth digit, and the medial forearm, with preserved sensation elsewhere. Reflexes were intact, and no Horner syndrome was observed. This pattern of motor and sensory involvement, extending beyond a single peripheral nerve distribution, was most consistent with medial cord involvement. A focused ultrasound evaluation did not demonstrate findings suggestive of hematoma or other compressive lesions.

An arm sling was provided for protection, and the patient was counseled regarding likely inadvertent medial cord involvement secondary to anesthetic spread from the PECS block. She was discharged home with instructions for close monitoring. Daily virtual follow-up was conducted until full resolution of symptoms, which occurred six days post-procedure. Despite the unintended block, the patient reported satisfactory analgesia at the surgical site, and no long-term complications were observed.

## Discussion

This case describes transient postoperative upper extremity neurologic symptoms associated with PECS I and II blocks. The sensory and motor findings predominantly affected a C8-T1 distribution, consistent with lower brachial plexus involvement, including medial cord participation. Although brachial plexus involvement following PECS blocks is uncommon, similar presentations have been reported in isolated cases [5,6].

**Figure 2:**
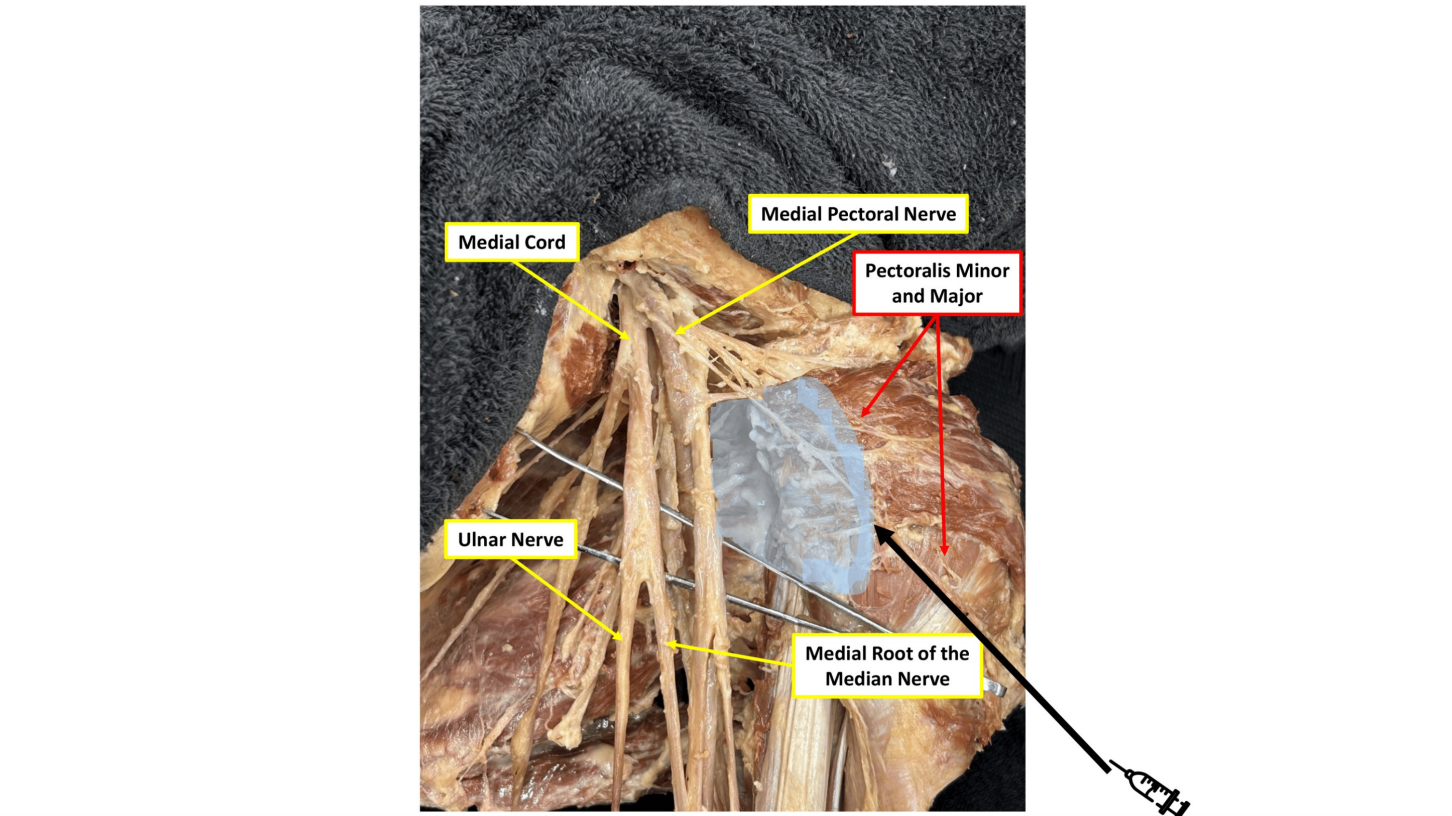
Infraclavicular cadaveric dissection showing anatomic relationship of medial cord branches to pectoral muscles Cadaveric dissection of the infraclavicular region demonstrating the medial cord of the brachial plexus and its branches, including the ulnar nerve, medial root of the median nerve, and medial PECS. The proximity of these structures to the pectoralis major and pectoralis minor muscles is shown, highlighting the potential pathway for unintended proximal spread of local anesthetic from the PECS I/II injection planes. The images were obtained from an adult human cadaver donated to the Rutgers New Jersey Medical School Anatomical Donation Program and used in accordance with institutional educational and ethical guidelines. PECS: pectoral nerve Image Credit: Authors, using Microsoft PowerPoint (Microsoft Corporation, Redmond, Washington, USA)

A possible mechanism may be related to the anatomical continuity between the interpectoral and axillary fascial planes. The interpectoral fascia, situated between the pectoralis major and minor muscles (Figure 1), is continuous with the clavipectoral fascia, which encloses the thoracoacromial vessels and the lateral PECS as they course toward the axilla. The lateral PECS (C5-C7) pierces the clavipectoral fascia to supply the pectoralis major, whereas the medial PECS (C8-T1) traverses the pectoralis minor before innervating both pectoral muscles (Figure 2) [7].

The PECS I block targets the interpectoral plane to anesthetize the medial and lateral PECS, while the PECS II block involves deposition of local anesthetic between the pectoralis minor and serratus anterior muscles. This latter plane lies adjacent to the clavipectoral fascia, providing a potential route for proximal spread of local anesthetic toward the axilla [1,7]. Given that both PECS arise from the brachial plexus, anesthetic tracking along these fascial planes may reach the cords, explaining the C8-T1 distribution deficits observed in our case.

Infraclavicular cadaveric studies have demonstrated that the integrity of fascial layers plays a critical role in limiting local anesthetic spread. In intact, pre-surgical anatomy, an injectate volume of approximately 10 mL per plane typically remains confined within the targeted fascial compartments, effectively anesthetizing the medial and lateral PECS without proximal extension to the brachial plexus cords [2,8]. Disruption of these fascial planes, such as that caused by surgical dissection, can permit more extensive diffusion of an anesthetic. In the present case, the patient underwent bilateral augmentation mammoplasty using a submuscular approach, which involves dissection of the pectoral plane and may alter local anesthetic spread by compromising fascial integrity. Importantly, the PECS I and II blocks were administered postoperatively, after surgical dissection had already disrupted normal fascial planes, which may have facilitated proximal diffusion of local anesthetic toward the medial cord [5]. In addition, the use of a relatively large injectate volume (15 mL per plane) may have further contributed to unintended proximal spread toward the medial cord. Increased pressure from the injectate may also have contributed to transient neuropraxia, which could help explain the relatively prolonged duration of medial cord involvement beyond the expected pharmacologic duration of local anesthetics. The relatively rapid recovery is less consistent with direct surgical or structural nerve injury, which typically results in more persistent deficits [9].

Although other causes of neuropraxia, including perioperative positioning-related injury, remain alternative considerations, such injuries, most commonly involving the ulnar nerve, typically present immediately after surgery and often follow a prolonged recovery course [9]. In contrast, the delayed onset several hours after surgery, the neurologic pattern suggestive of lower brachial plexus involvement rather than isolated ulnar nerve compression, and complete resolution within six days observed in this case are less characteristic of classic positioning-related neuropraxia. Other potential etiologies, including medial cord stretch injury, compressive lesions such as postoperative hematoma, local anesthetic systemic toxicity (LAST), and inadvertent intraneural injection, were also considered. However, stretch injuries are typically associated with intraoperative positioning factors such as excessive arm abduction, shoulder external rotation, or prolonged traction, and usually present immediately postoperatively with more prolonged deficits, which were not observed. Compressive lesions would be expected to produce progressive symptoms and local signs such as swelling, pain, or mass effect; additionally, post-procedural ultrasound did not demonstrate findings suggestive of a compressive lesion [10]. LAST presents with systemic neurologic or cardiovascular manifestations, whereas intraneural injury would more likely result in persistent, focal deficits rather than a patchy distribution pattern [11]. Taken together, these findings support a multifactorial mechanism while acknowledging that definitive attribution remains uncertain.

This case emphasizes that although PECS I and II blocks are generally safe, medial cord involvement can occur as a rare but clinically significant and underrecognized complication. Understanding the relevant anatomy, using conservative injectate volumes, and maintaining awareness of potential fascial disruption are key to minimizing this risk. Additionally, this case contributes to the limited literature on brachial plexus involvement following PECS blocks and supports the hypothesis that altered fascial integrity and high-volume injections may facilitate proximal anesthetic spread.

## Conclusions

This case highlights an uncommon but clinically meaningful complication of PECS I and II blocks: unintended medial cord involvement resulting in transient C8-T1 motor and sensory deficits. This report reinforces that local anesthetic spread beyond the intended fascial planes can occur, particularly in the setting of higher injectate volumes and potential surgical disruption of fascial integrity. While the temporal relationship and clinical pattern support block-related proximal anesthetic spread as the most plausible explanation, definitive causation cannot be established. A thorough understanding of the clavipectoral and axillary fascial anatomy, careful selection of injectate volume, and heightened vigilance for postoperative neurologic symptoms are essential to mitigate this risk. Prompt recognition and conservative management can lead to full recovery without long-term sequelae, while preserving the analgesic benefits of PECS blocks. This case further characterizes brachial plexus involvement associated with PECS blocks and underscores the importance of ongoing anatomical and technical refinement in regional anesthesia practice.

## References

[1] Bin Ghali K, AlKharraz N, Almisnid O, Alqarni A, Alyamani OA (2023). The pectoral (PECS) regional block: a scoping review. Cureus.

[2] Desroches J, Grabs U, Grabs D (2013). Selective ultrasound guided pectoral nerve targeting in breast augmentation: How to spare the brachial plexus cords?. Clin Anat.

[3] Gagnier JJ, Kienle G, Altman DG, Moher D, Sox H, Riley D (2013). The CARE guidelines: consensus-based clinical case reporting guideline development. Headache.

[4] EQUATOR Network (2026). The CARE guidelines: consensus-based clinical case reporting guideline development. https://www.equator-network.org/reporting-guidelines/care/.

[5] Mathers JD, Engum A, Galleberg G (2023). Brachial plexus blockade arising from a combined pectoralis (PECS) 1 and 2 block. Anaesth Rep.

[6] Kulkarni M, Diwan S, Nair A (2020). Failure of PECS 2 block and a numb hand!!. Saudi J Anaesth.

[7] Kelava M, Alfirevic A, Bustamante S, Hargrave J, Marciniak D (2020). Regional anesthesia in cardiac surgery: an overview of fascial plane chest wall blocks. Anesth Analg.

[8] Elshanbary AA, Zaazouee MS, Darwish YB (2021). Efficacy and safety of pectoral nerve block (PECS) compared with control, paravertebral block, Erector spinae plane block, and local anesthesia in patients undergoing breast cancer surgeries: a systematic review and meta-analysis. Clin J Pain.

[9] Hewson DW, Bedforth NM, Hardman JG (2018). Peripheral nerve injury arising in anaesthesia practice. Anaesthesia.

[10] Kim HJ, Park SH, Shin HY, Choi YS (2014). Brachial plexus injury as a complication after nerve block or vessel puncture. Korean J Pain.

[11] Goyal R, Shukla RN (2012). Local anesthetic systemic toxicity (LAST) - should we not be concerned?. Med J Armed Forces India.

